# Novel Use of Folate-Targeted Intraoperative Fluorescence, OTL38, in Robot-Assisted Laparoscopic Partial Nephrectomy: Report of the First Three Cases

**DOI:** 10.1089/cren.2016.0104

**Published:** 2016-11-01

**Authors:** Cheuk Fan Shum, Clinton D. Bahler, Philip S. Low, Timothy L. Ratliff, Steven V. Kheyfets, Jay P. Natarajan, George E. Sandusky, Chandru P. Sundaram

**Affiliations:** ^1^ Department of Urology, Indiana University School of Medicine, Indianapolis, Indiana.; ^2^ Department of Chemistry, Institute for Drug Discovery, Purdue University, West Lafayette, Indiana.; ^3^ Center for Cancer Research, Purdue University, West Lafayette, Indiana.; ^4^ Department of Pathology and Laboratory Medicine, Indiana University School of Medicine, Indianapolis, Indiana.

**Keywords:** OTL38, folate-targeted intraoperative fluorescence, partial nephrectomy

## Abstract

Partial nephrectomy is now the preferred surgical option for small renal tumors because it allows nephron preservation without compromising oncologic clearance. Its outcomes depend on the surgeon's ability to continuously identify the edges of the tumor during resection, thus leaving an adequate margin around the tumor without excessive removal of normal parenchyma, as well as keeping a short ischemic time. Folate receptors are highly abundant in the normal kidney, and there is a difference in folate receptor expression between malignant and normal renal tissues. Thus, the use of fluorescent agents that target folate receptors should result in differential fluorescence between the tumor and surrounding parenchyma during partial nephrectomy, which, in turn, helps tumor demarcation for identification and resection. A phase 2 study on the novel use of OTL38 in robot-assisted laparoscopic partial nephrectomy is currently in progress in our institution. The outcomes of the first three cases have shown the possible advantages of OTL38 in intraoperative tumor identification before resection and recognition of residual disease in the surrounding parenchyma after resection. The tumors typically appeared dark while the surrounding parenchyma showed brighter fluorescence. Immediately after tumor resection, the margins of all the specimens appeared to have a uniformly bright fluorescence, suggestive of an intact margin of normal renal parenchyma along the plane of excision. The pattern of intraoperative fluorescence correlates well with immunohistochemistry. No OTL38-related adverse effects have been seen among these three patients. We present the outcomes of these three cases, illustrated with intraoperative and immunohistochemistry images.


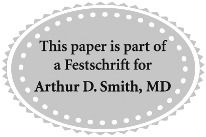


## Introduction

Both partial nephrectomy and radical nephrectomy for small renal tumors show similar oncologic outcomes.^1,2^ With increasing evidence showing poorer renal function and negative cardiovascular effects after radical nephrectomy, the role of partial nephrectomy as the first-line surgical option is widely accepted.^2,3^ Well-performed partial nephrectomies should include two routine criteria: a negative margin without excessive removal of surrounding normal parenchyma and a short warm ischemic time. Various factors have an impact on the outcomes of partial nephrectomy, including surgical approach, surgeons' experience, and the availability of new technologies to improve each critical step during surgery.^4,5^

Besides three-dimensional laparoscopic vision and added dexterity, the use of robotic assistance in partial nephrectomy has the added advantage of having various imaging and filter systems to possibly help with visual demarcation of tumors from normal renal parenchyma.^6,7^ One such example is near-infrared fluorescence imaging. It involves the activation of an exogenous fluorescent tracer resulting in light emission in the near-infrared spectrum, which is then captured by a built-in charge-coupled device camera.^8,9^ The differential fluorescent glow in the tumor and normal renal parenchyma aids tumor excision with a margin and detection of residual tumors in the surgical field. There are many exogenous fluorescent tracers that are suitable for near-infrared fluorescence imaging, with the most widely studied being indocyanine green.^8^

Folate targeting involves the use of folate analogs to deliver imaging and therapeutic agents to tissues with a high concentration of folate receptors.^10^ It has been found that folate receptors are highly abundant in the normal kidney, and there is a difference in folate receptor expression between malignant and normal renal tissues.^11,12^ The use of a folate analog conjugated to a fluorescent tracer during partial nephrectomy may therefore help to identify the local extent of the tumor immediately before surgical excision. The concept of folate-targeted intraoperative fluorescence has been applied effectively in the surgical management of ovarian cancer.^13^ Folate receptors are highly expressed in ovarian tumors but minimally within normal ovarian tissues, resulting in the intraoperative finding of brightly fluorescent tumors surrounded by normal tissues without fluorescence. In contrast, folate receptors are highly expressed in normal kidneys, but less so within renal tumors. Therefore, the pattern of intraoperative fluorescence in partial nephrectomy may be different from that in ovarian cancer surgery.

We are currently performing a phase 2 nonrandomized study on the novel use of folate-targeted intraoperative fluorescence in renal cancer, and we are reporting the outcomes of the first three cases of partial nephrectomy in this ongoing study.

## Materials and Methods

The institutional review board approved this study (protocol number: 1511879268, approval date: December 16, 2015). The entire phase 2 study involves a total of 20 patients in 2 arms, with 10 having localized renal-cell carcinoma (RCC) for robot-assisted laparoscopic partial nephrectomy (RALPN) and 10 having locally advanced or metastatic RCC for open or robot-assisted laparoscopic radical nephrectomy. The main objective is to explore the use of folate-targeted intraoperative fluorescence to identify the margins of resection in RALPN and to identify lymph nodes or other metastases in radical nephrectomy. For the purpose of this article reporting the outcomes of the first three cases of RALPN, we shall elaborate on the study protocol in the RALPN arm. Table 1 illustrates the inclusion and exclusion criteria.

**Table 1.** Inclusion and Exclusion Criteria for the Partial Nephrectomy Arm of the Study

*Inclusion criteria*	*Exclusion criteria*
At least 18 years old	Known anaphylactic or allergic reactions to folate and its analogs
Proven or suspected diagnosis of cT1-2 RCC by CT assessment	Known sensitivity to fluorescent light
Scheduled for partial nephrectomy	Renal impairment (baseline GFR below 50 mL/min/1.73 m^2^)
Expected survival of at least 3 months	Hepatic disorders (CTCAE version 4 definition ≥ grade 2)
Good performance status (ECOG ≤1)	Brain metastases
Negative serum or urine pregnancy test within 24 hours before partial nephrectomy (for females of child-bearing age)	Participation in another investigational drug trial within 30 days before partial nephrectomy
Willingness to participate after the process of informed consent	Any medical conditions that, in the opinion of the investigators, could potentially jeopardize patient's safety, limit the patient's ability to complete the study, or compromise the objectives of the study

CTCAE = Common Terminology Criteria for Adverse Events; ECOG = Eastern Cooperative Oncology Group; GFR = glomerular filtration rate; RCC = renal-cell carcinoma.

Within 90 days before surgery, the patients attend a screening visit, during which they give informed consent for the study. Their demographics, height and weight, medical history, and physical examination findings are recorded. Complete blood count, serum electrolyte levels, and renal function are also measured.

On the day of surgery, a single dose of OTL38 (On Target Laboratories LLC., West Lafayette, IN) at 0.025 mg/kg and a single dose of 25 mg diphenhydramine are administered intravenously within 2 hours before skin incision. OTL38 is constructed from folic acid linked by its γ-carboxyl and a short spacer to an indocyanine green-related near-infrared dye termed SO456.^14^ It targets folate receptor alpha (FRα), which is highly expressed in the kidney. Upon excitation, it emits light in the near-infrared spectrum. During RALPN, the da Vinci^©^ Fluorescence Imaging Vision System (Intuitive Surgical, Inc., Sunnyvale, CA) is used to identify tumor margins with visible and fluorescent light immediately before excision and to identify any residual disease immediately after excision. Video and still images are taken in visible and fluorescent light immediately before and after excision. Upon excision, the tumor specimen is sent to the pathologist for inking and sectioning into 5-mm-thick slices for macroscopic assessment of the margin status. Again, video and still images of the tumor specimen in visible and fluorescent lights are taken.

At the end of surgery, the surgeon completes a questionnaire describing the usefulness of folate-targeted intraoperative fluorescence for tumor identification and excision. A coinvestigator, who is a blinded surgeon uninvolved with the case, views the visible and fluorescent light images and completes a questionnaire to assess interobserver agreement. Any adverse effects related to the use of OTL38, such as hypersensitivity or injection site inflammatory reactions, are noted.

**FIG. 1.**
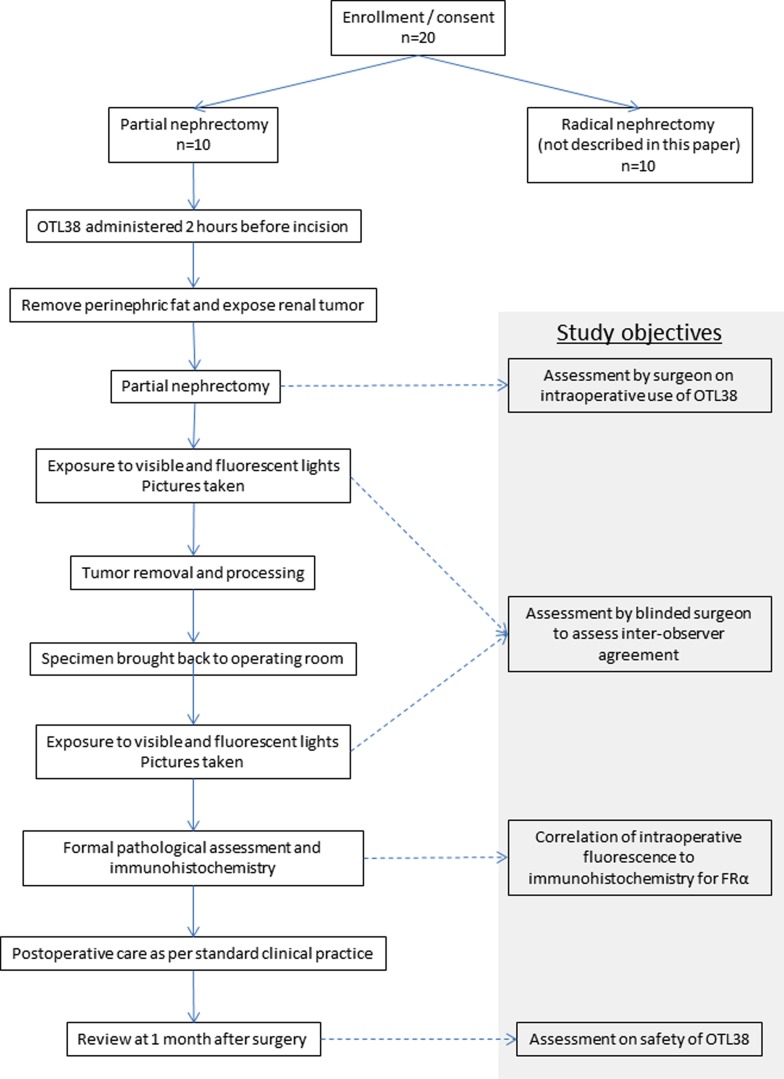
Study protocol on the use of OTL38 in robot-assisted laparoscopic partial nephrectomy.

Postoperatively, the patients are monitored as per standard clinical practice. Serial assessments with complete blood counts, serum electrolytes, and renal functions are performed. A review visit at 1 month after surgery provides an eventual assessment of surgical outcomes and identifies any delayed adverse effects from the use of OTL38. The final histopathology of the tumor specimen is also noted. Figure 1 summarizes the study protocol on the use of OTL38 in RALPN.

The primary objective of this study is to explore the use of OTL38 during RALPN and the pattern of fluorescence in the tumor and surrounding parenchyma. The secondary objectives include correlation between intraoperative tumor fluorescence and FRα status of tumor specimens by immunohistochemistry, assessment of the usefulness of OTL38 in intraoperative identification of tumor margins by the operating surgeon, assessment of any interobserver variations by a blinded coinvestigator, and assessment of the clinical safety of OTL38 among RCC patients up to 1 month after surgery.

## Results

So far, three patients have completed treatment and follow-up as per study protocol in the RALPN arm in this ongoing study.

**FIG. 2.**
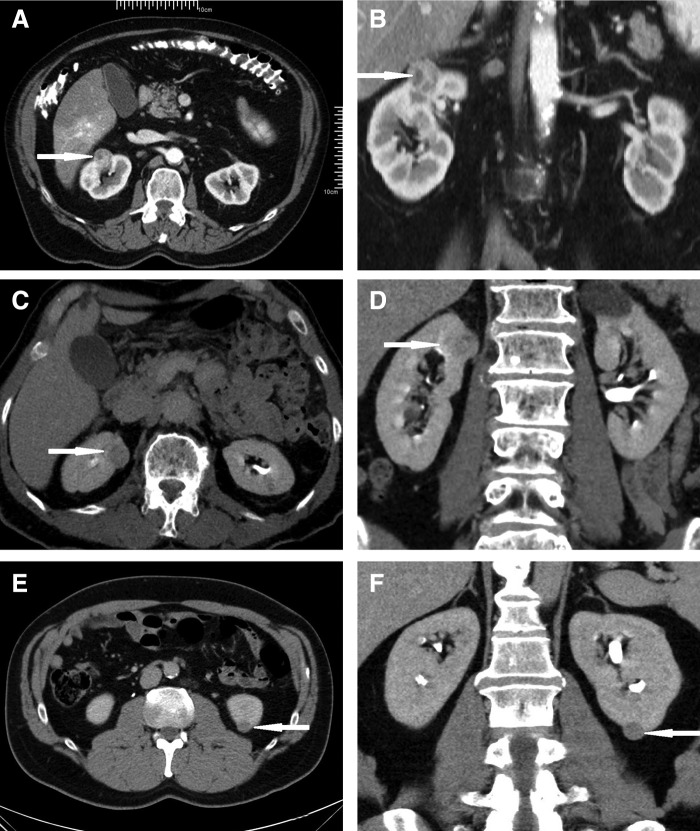
CT images of the patients, with tumors indicated by *arrows*. **(A, B)** Transverse and coronal views of renal tumor in the first patient. **(C, D)** Transverse and coronal views of renal tumor in the second patient. **(E, F)** Transverse and coronal views of renal tumor in the third patient.

All three patients were men and ranged from 50 to 70 years of age. They presented with incidental small renal masses 15 to 22 mm in largest diameter. Figure 2 shows the transverse and coronal CT images representative of their renal masses. All patients received OTL38 and underwent RALPN as per study protocol. Fluorescent lights were used to identify tumor margins immediately before excision and to look for possible residual disease in the resection bed immediately after excision. All tumors were excised by sharp dissection with scissors under visible light and near-infrared imaging. A two-layered renorrhaphy was routinely performed for hemostasis and repair of the defect after excision in all three patients. The mean length of hospital stay was 2 days (range: 1–3 days).

**FIG. 3.**
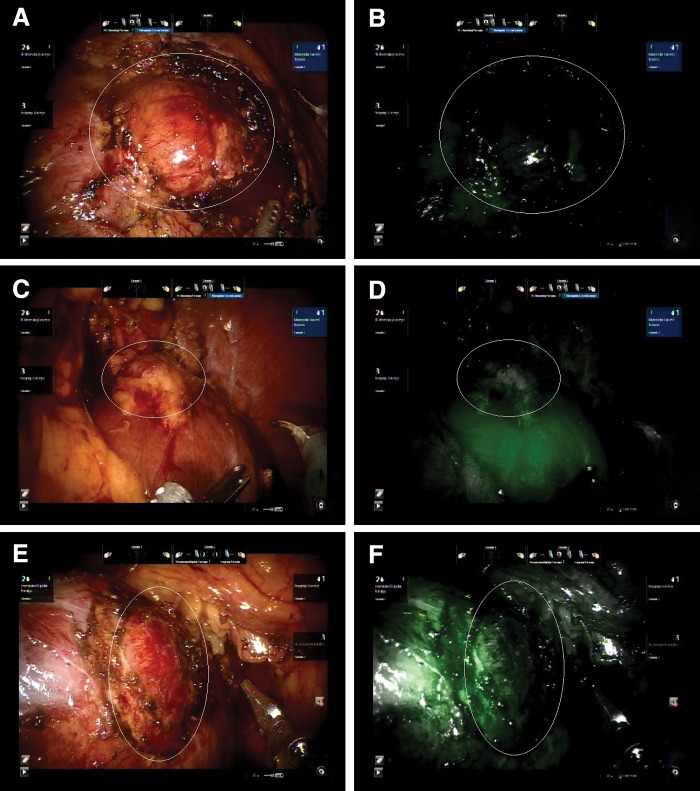
Pattern of intraoperative fluorescence of the patients. Tumors are highlighted with *oval white lines*. **(A, B)** Renal tumor and surrounding parenchyma of the first patient under visible and fluorescent lights. **(C, D)** Renal tumor and surrounding parenchyma of the second patient under visible and fluorescent lights. **(E, F)** Renal tumor and surrounding parenchyma of the third patient under visible and fluorescent lights.

The pattern of intraoperative fluorescence among all three patients was similar. All renal tumors did not appear fluorescent, whereas the surrounding parenchyma showed mild fluorescence in the first patient and bright fluorescence in the second and third patients. Figure 3 shows the pattern of intraoperative fluorescence in the patients.

The complete lack of fluorescence in the renal tumors surrounded by fluorescent parenchyma served as a good indicator for the tumor margins in all the three patients. During excision of the tumors, intermittent switching from visible to fluorescent light also helped in confirming the correct depth of excision. Warm ischemic time, measured from the clamping of the renal arteries to the release of clamps after the first layer of renorrhaphy, ranged from 10 to 16 minutes. The estimated blood loss ranged from 50 to 200 mL.

**FIG. 4.**
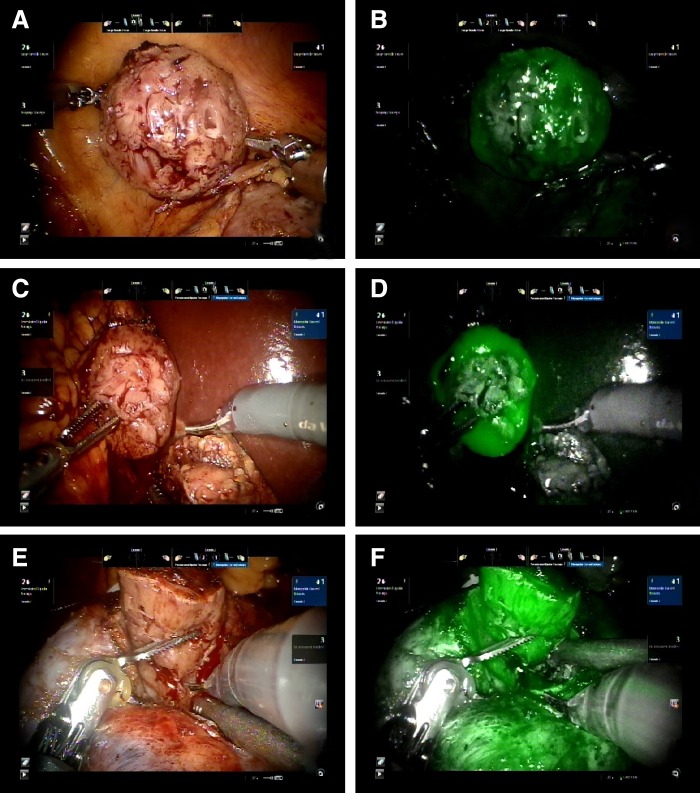
Cut surfaces of the tumors immediately after excision. **(A, B)** Visible and fluorescent light images of the first patient. **(C, D)** Visible and fluorescent light images of the second patient. **(E, F)** Visible and fluorescent light images of the third patient.

Immediately after tumor excision, the margin of the specimen appeared to have a uniformly bright fluorescence, suggestive of an intact margin of normal renal parenchyma around the plane of excision. Figure 4 shows the cut surfaces of the tumors under visible and fluorescent light.

**FIG. 5.**
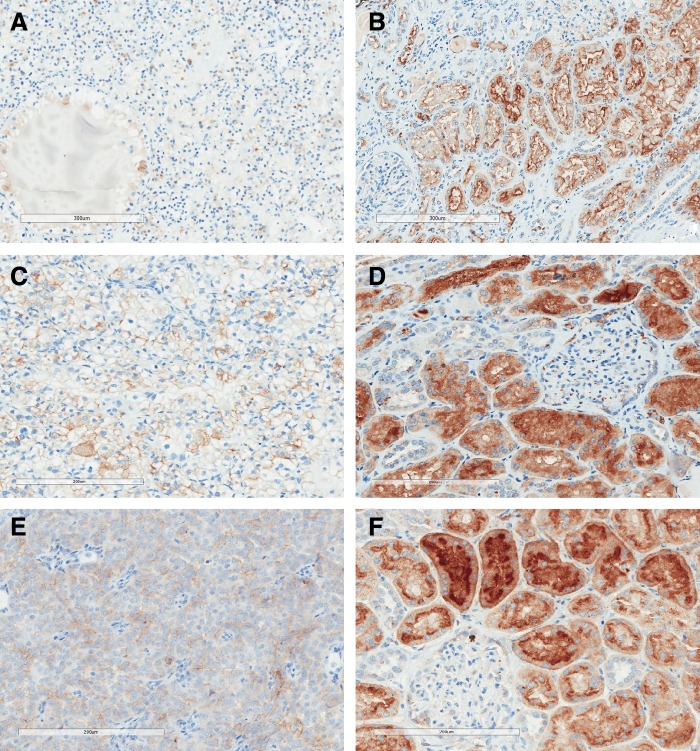
Immunohistochemistry of the three patients showing consistently strong staining for FRα in normal renal parenchyma, but minimal staining in RCC. **(A, B)** Staining for FRα in tumor and renal parenchyma of the first patient. **(C, D)** Staining for FRα in tumor and renal parenchyma of the second patient. **(E, F)** Staining for FRα in tumor and renal parenchyma of the third patient. FRα, folate receptor alpha; RCC, renal-cell carcinoma.

Upon histologic examination of the tumor specimens, the first and second patient showed Fuhrman grade 2 clear-cell RCC with uninvolved margins, and the third patient showed Fuhrman grade 2 type 1 papillary RCC with uninvolved margins. Immunohistochemistry showed consistent correlation with the pattern of intraoperative fluorescence in all the three patients. Only 10% to 30% of tumor cells were stained for FRα, but the surrounding renal parenchyma demonstrated strong staining, especially along the luminal surface of the renal tubular epithelium. Figure 5 shows the immunohistochemistry results of the patients.

All the three patients had an uneventful postoperative recovery and did not show any OTL38-related adverse effects up to 1 month after the surgery. There were no significant perioperative changes in serum electrolyte levels and renal function. Table 2 summarizes the demographics and outcomes of our three patients.

**Table 2.** Summary of Demographics and Outcomes of the Patients

*Patient*	*Age (year)*	*BMI*	*ECOG*	*Tumor size (mm)*	*R.E.N.A.L. nephrometry score*	*Fluorescence in tumor*	*Fluorescence in parenchyma*	*Usefulness of OTL38 in tumor identification and excision*	*Warm ischemic time (minutes)*	*Estimated blood loss (mL)*	*OTL38-related adverse effects*	*Histology*	*FRα staining in tumor*	*FRα staining in parenchyma*
1	67	31.6	0	22	1+1+2+A+2	Nil	Mild	Yes	13	200	Nil	Fuhrman grade 2 clear-cell RCC, margins uninvolved	Minimal intensity in <10% tumor cells	Strong
2	70	24.2	0	22	1+2+3+P+1	Nil	Bright	Yes	16	200	Nil	Fuhrman grade 2 clear-cell RCC, margins uninvolved	Mild intensity in <30% tumor cells	Strong
3	50	33.2	0	15	1+1+1+P+1	Nil	Bright	Yes	10	50	Nil	Fuhrman grade 2 papillary RCC (type 1), margins uninvolved	Mild intensity in 50% of tumor cells	Strong

BMI = body mass index.

## Discussion

All forms of cancer surgery with the intent to cure should focus on oncologic clearance, and partial nephrectomy for renal tumors is no exception. Partial nephrectomy is advantageous over radical nephrectomy for small renal tumors because of nephron preservation, yet it remains noninferior in terms of oncologic outcomes.^1,2^ Various modalities are used to increase the negative margin rates during partial nephrectomy. These include intraoperative imaging such as ultrasonography, pathologic assessments such as frozen section, and the use of fluorescent tracers such as indocyanine green to pinpoint the tumor edge.^7,9,15–19^ When used singly or in combination, these modalities provide intraoperative assurance to the surgeon that tumor resection is complete and residual disease is unlikely. The ability to continuously and accurately identify the tumor margin during partial nephrectomy also reduces unnecessary pauses to confirm the plane of resection, thus potentially avoiding prolonged arterial clamping and warm ischemia. Warm ischemic time has a direct impact on postoperative renal function which, in turn, correlates with cardiovascular complications and overall survival.^20^

Advances in biomolecular research have also contributed to improved outcomes of cancer surgery. The expressions of FRα in various human cancer and normal tissues have been delineated.^11,12^ There are differences in expression between cancer and normal tissues in several organs, including ovary and kidney. Such discrepancies in FRα expression can be exploited clinically, such that folate analogs conjugated to various contrast agents show different degrees of binding to normal and malignant tissues. The recent use of folate-targeted intraoperative fluorescence with OTL38 among patients with ovarian cancer has shown encouraging results, allowing resection of an additional 29% of malignant lesions that were previously unidentifiable by inspection or palpation.^13^ Ovarian tumors and metastatic deposits appeared fluorescent and were easily located during surgery.

In the human kidney, FRα expression reaches 100% at the apical surface of the proximal tubules, where it plays the physiologic role of folate reabsorption.^21^ Renal carcinoma, whether *in vivo* or in established cell lines, shows a lower expression of FRα.^11,12^ The pattern of intraoperative fluorescence in our three patients appeared to be consistent with the difference in FRα expression in normal and malignant renal tissues and facilitated the identification of the local extents of the tumors. The bright fluorescence along the cut surfaces of the tumors at the end of resection could be intuitively recognized by the surgeon as an indication of adequate margin. While the study is still ongoing, such initial findings seem to indicate that folate-targeted intraoperative fluorescence with OTL38 is feasible and facilitates the recognition and resection of small tumors in partial nephrectomy. Upon completion of the entire phase 2 study, we shall be able to report the negative margin rate, warm ischemic time, and other operative parameters associated with the use of OTL38.

The choice of FRα-targeting fluorescent tracer for partial nephrectomy should be carefully considered. The difference in fluorescence between renal parenchyma and tumor should be as distinct as possible. In our three cases of partial nephrectomy where the tumors had no fluorescence, bright fluorescence in the renal parenchyma would be ideal. We had access to two tracers, namely EC17 and OTL38, and both contain the same folate ligand for binding with FRα. They differ in the fluorochrome, with EC17 carrying fluorescein, which fluoresces with a peak wavelength of 510 nm, and OTL38 carrying indocyanine green, which fluoresces with a peak wavelength of 794 nm.^14^ Comparison between the two tracers showed that OTL38 has the advantages of deeper tissue penetration, reduced scattering, and reduced autofluorescence. Most importantly, OTL38 fluoresces in the kidneys, whereas EC17 does not.^14^ Previous experience with EC17 for partial nephrectomy had problems with autofluorescence and poor tissue penetration, and tumor fluorescence was only present in 50% of cases.^22^ Therefore, OTL38 seemed to be a more suitable tracer.

Immunohistochemistry results from our three patients confirmed significantly lower expression of FRα in tumors than in normal parenchyma. Both the intensity and the density of stains were much lower within tumors, consistent with the observed difference in intraoperative fluorescence between normal and malignant renal tissues. Such immunohistochemistry also concurs with the findings from previous studies using polymerase chain reaction or radioligand binding assay to determine FRα expression in frozen human tissues and established cell lines.^11,12^ With two of our patients having clear-cell RCC and one having papillary RCC, it seems that both histologic cell types are poor in FRα expression compared with normal renal parenchyma.

Interestingly, while our patients demonstrated poor expression of FRα in the small localized renal tumors, a previous study showed much higher expression of folate receptors in metastatic RCC. Using an imaging agent that consisted of folate conjugated to technetium-99m, 74% of metastatic RCC showed mild to marked uptake.^23^ The difference in folate receptor expression between small localized renal tumors and metastatic RCC raises the possibility for folate targeting to be used for prognostication in RCC.

Based on the current medical literature, the most common OTL38-related adverse event is hypersensitivity reaction, and its occurrence seems to be dose related.^13^ We chose the dose of 0.025 mg/kg after reviewing safety and efficacy data from previous studies.^13,24^ The concurrent administration of an antihistamine agent was intended to further reduce the risk of hypersensitivity reactions among our study patients. There were no OTL38-related adverse events observed in all of our patients for up to a month after the surgery. However, with outcomes from only three patients, we are unable to comment on the clinical safety of OTL38 among patients with renal tumors at the current moment.

## Conclusion

The outcomes of the initial three patients seemed encouraging, with easy tumor identification and resection due to the clearly observed difference in intraoperative fluorescence in renal tumors and their surrounding renal parenchyma. We also demonstrated a close correlation between intraoperative fluorescence with OTL38 and FRα staining on immunohistochemistry. As more patients complete the study, we hope to draw more definite conclusions on the use of OTL38 for RALPN, including its intraoperative fluorescence pattern, impact on warm ischemic time and negative margin rate, and adverse effects. We also hope to confirm if these outcomes may be affected by other factors, such as tumor cell types and other patient-dependent variables.

## Abbreviations Used

BMI: body mass index
CT: computed tomography
CTCAE: Common Terminology Criteria for Adverse Events
ECOG: Eastern Cooperative Oncology Group
FRα: folate receptor alpha
GFR: glomerular filtration rate
RALPN: robot-assisted laparoscopic partial nephrectomy
RCC: renal-cell carcinoma
